# The Human Gastrointestinal Tract, a Potential Autologous Neural Stem Cell Source

**DOI:** 10.1371/journal.pone.0072948

**Published:** 2013-09-04

**Authors:** Cornelia Irene Hagl, Sabine Heumüller-Klug, Elvira Wink, Lucas Wessel, Karl-Herbert Schäfer

**Affiliations:** 1 Clinic of Pediatric Surgery, Medical Faculty Mannheim, University of Heidelberg Mannheim, Germany; 2 Life Science Department, Faculty of Computer Sciences and Microsystems Technology, University of Applied Sciences, Zweibrücken, Germany; University of Nebraska Medical Center, United States of America

## Abstract

Stem cell therapies seem to be an appropriate tool for the treatment of a variety of diseases, especially when a substantial cell loss leads to a severe clinical impact. This is the case in most neuronal cell losses. Unfortunately, adequate neural stem cell sources are hard to find and current alternatives, such as induced programmed stem cells, still have incalculable risks. Evidence of neurogenesis in the adult human enteric nervous system brought up a new perspective. In humans the appendix harbors enteric neuronal tissue and is an ideal location where the presence of neural stem cells is combined with a minimal invasive accessibility. In this study appendices from adults and children were investigated concerning their neural stem cell potential. From each appendix tissue samples were collected, and processed for immunohistochemistry or enteric neural progenitor cell generation. Free-floating enteric neurospheres (EnNS’s) could be generated after 6 days *in vitro*. EnNS’s were either used for transplantation into rat brain slices or differentiation experiments. Both transplanted spheres and control cultures developed an intricate network with glia, neurons and interconnecting fibers, as seen in primary enteric cultures before. Neuronal, glial and neural stem cell markers could be identified both *in *vitro and *in vivo* by immunostaining. The study underlines the potential of the enteric nervous system as an autologous neural stem cell source. Using the appendix as a potential target opens up a new perspective that might lead to a relatively unproblematic harvest of neural stem cells.

## Introduction

Stroke, cerebrovascular disease and neurodegeneration are major causes of mortality and disability [1]. Their common characteristics are a progressive loss of structure and function [2] that cannot be overcome by healing processes. Despite dramatic advancements in medical care, effective clinical therapies are limited and therapeutic strategies that restore neuronal and glial cells as well as functional circuits are lacking. Neurodegeneration predominantly affects specific neuronal populations, like dopaminergic neurons in Parkinson’s disease [3] or cortical and hippocampal neurons in Alzheimer’s disease [4]. In brain injury the affected area comprises a wide range of neuronal and connective tissues [2], [5]. Furthermore the regenerative processes have both beneficial and detrimental effects. Surrounding glial cells even contribute to the pathologic processes by forming a glial scar [6], [7] or supporting neurotoxicity in neurodegenerative diseases [8]. Therapeutic strategies therefore should facilitate endogenous regeneration, attenuate neurotoxic effects and achieve functional improvement. Stem cell transplantation therefore has emerged as a potential therapeutic approach for cell replacement in ischemic brain injuries or neurodegenerative diseases [9]–[11].

Therapeutic studies with embryonic tissue, autologous cell sources and mesenchyme or induced pluripotent stem cells still have their drawbacks [12]–[14]. The potential tumorigenicity of transplants derived from embryonic stem cells, ethical concerns and a limited availability of standardized, viable and pure embryonic stem cells make it unlikely that embryonic stem cell transplantation will become a routine treatment in the near future [15]. Autologous cell sources circumvent ethical problems and the need for immunosuppression but depend on appropriate sources, which yield sufficient amounts of donor cells in appropriate quality. The discovery of neural stem cell niches in the adult brain has raised the possibility of endogenous neuronal replacement for neuronal tissue repair. But in the normal human brain, progenitor cells in the subventricular zone (SVZ) are present in relatively low abundance and the tissue damage might also affect the neurogenic niches. Moreover, to isolate sufficient amounts of donor tissue from the SVZ is a risk in itself and might destroy the niche properties. In particular, many stroke patients have infarcts located close to the SVZ [16] and a number of studies showed changes in the amount of proliferating cells in the human brain SVZ in Parkinson’s (PD) and Alzheimer’s disease (AD) [9], [17].

Mesenchyme and induced pluripotent stem cells (iPSC’s) are a promising source of multipotent self renewing cells but have to be redifferentiated into lines with complex neuronal diversity (e.g. cholinergic or dopaminergic neurons) before transplantation [18]. But the programming and differentiation of iPSC’s is challenging and they also carry the risk of tumorgenesis or cytogenetic abnormalities as a result of extensive *in vitro* manipulation. To precede stem cell transplantation into a feasible treatment for neuronal tissue loss therapeutic approaches must combine a neural stem cell source that persists into adulthood with an easy and minimal invasive access. Ideally the stem cell niche contains progenitors, which maintain their plasticity and differentiation potential in culture [19]. The transplanted neural precursors may then assimilate and constitute a neural network similar to that lost in disease [20]. Alternatively, stem cells might provide environmental enrichment to facilitate host neurons by producing neurotrophic factors, attenuate toxic influences, or create supplementary neuronal networks around affected tissues [21]–[24].

For central nervous system (CNS) repair the gastrointestinal tract with its neural crest derived neuronal and glial cells; the enteric nervous system (ENS) harbours a potent and easily accessible autologous source [25], [26]. Moreover, due its similarities with the central nervous system (CNS) such as its complex neuronal organization, the neurotransmitter spectrum and chemical coding [27] the ENS is a excellent neural stem cell source for cell replacement therapies. Neural crest derived stem cells are responsible for the plasticity in the ENS, which is to be seen all over its life span [28]. Autologous cell therapy for neurological and intestinal disorders has to be advanced, and enteric stem cells may become the ideal candidate for neural cell transplantation. Neural stem cells isolated from the ENS proliferate and form *in vitro* neurospheres that maintain both the capability of self renewal and the ability to differentiate into neurons and astrocytes [28]–[30]. The appendix, as an abdicable part of the intestine, might be the appropriate location with a sufficient amount of enteric nervous tissue [31] where neural stem cells are easily harvested.

We therefore used appendices from patients at different ages to cultivate and differentiate enteric neurospheres from adult human tissue. The cultivated enteric neurospheres (EnNS’s) demonstrated specific stem cell characteristics as confirmed by immunofluorescence staining and qPCR, while neuronal and glial cells could be derived from these EnNS’s. To proof the hypothesis, that these enteric neural cells are a possible source for CNS tissue replacement a pilot study was performed. To proof its feasibility EnNS’s derived from postnatal intestinal tissue were transplanted into rat brain slices and investigated concerning their behavior. In parallel, neurospheres from newborn rats were generated routinely to establish and improve the transplantation procedures. During the observation period the neuronal and glial precursor cells survived, migrated and differentiated while constituting an intricate neuronal network inside the brain slices. In conjunction with various neuronal phenotypes, like catecholaminergic neurons in the EnNS’s this autologous neural stem cell treatment might be a feasible, minimally invasive, safe, and effective approach for neuronal cell replacement even in brain injury and neurodegenerative disease.

## Materials and Methods

### Animal Model

All tissues (whole intestine and brain slices) were dissected from eight days old (p8) Sprague Dawley (SD) rats.

This study was carried out in strict accordance with the recommendations for the care and use of laboratory animals of the German animal protection law. The experiments were performed after killing the animal by decapitation to minimize suffering, and only based on the post mortem removal of tissue. Following §6 of the German animal law, the tissue removal has only to be indicated. The study was approved by the local ethical committee *(Med. Ethik-Kommission II, Medical Faculty Mannheim).*

### Human Postnatal Appendices

Appendices from children and adults (age ranged from 6 month to 69 years) with either appendicitis or tumor resection were obtained from two surgical centers. A total of 81 tissue samples were included in the study. All samples were collected after written informed consent of the patients or the parents, following the Helsinki declaration and with the approval of the local ethical committee *(Med. Ethik-Kommission II, Medical Faculty Mannheim, 2010–352N-MA)*.

### Generation of Rat Enteric Neurospheres (rEnNS’s)

Briefly, rats were decapitated, the whole intestine removed and immediately stored in MEM (Gibco) on ice. The muscle and submucous layer were separated, and the dissected muscle tissue incubated in a collagenase II solution (Worthington) for 2 hours. After vortexing the tissue for 20 seconds the suspension was filtered through a sterile syringe filter (Cell strainer, BD biosciences), pore size 40 µm. The supernatant was centrifuged three times, the obtained myenteric plexus incubated in accutase *(PAA Laboratories GmbH)* for 20 minutes and dissociated by trituration. In a second centrifugation step, the accutase was removed and replaced with 10 ml culture medium. The cells were kept at standardized densities (1×10^6^ cells/25 cm^2^ flask) in culture medium adapted for expansion cultures [Neurobasal A (Gibco) supplemented with 2% B27- (without vitamin A, Gibco), 1% albumin (Sigma), 0.25% -2-mercaptoethanol (50 mM, Invitrogen), 0.12% glutamine (200 mM, Sigma), EGF (10 ng/ml, Tebue), bFGF (20 ng/ ml; Tebue), GDNF (10 ng/ml, Tebue), *gentamycin and metronidazole (100 µg/* *ml)*]. After two days in vitro, the first floating neurospheres (EnNS’s) were seen, while neurons and radiating glial cells expanded at the bottom.

### Generation of Human Enteric Neurospheres (huEnNS’s)

The tissues were stored in MEM-Hepes (Gibco) on ice immediately after surgery. Tissue samples for immunohistochemistry were removed and the remaining tissue processed for the isolation of human ENS’s. To generate huEnNS’s we modified the method published for embryonic gut tissue [32]. After separation of the submucous and muscular layer the tissue was enzymatically digested with *collagenase II solution (Worthington) for 4 hours* as described above for the rat intestine. Pure myenteric (MP) and submucous plexus (SMP) were isolated until all available enteric tissue could be obtained. The collected ganglia were mildly dissociated with accutase (PAA Laboratories GmbH) for 20 min and plated in 25 cm^2^ culture flasks (Nunc) at standardized densities (1×10^6^ cells/flask) using a standard neuronal medium [Neurobasal A (Gibco) supplemented with 2% B27- (without vitamin A, Gibco), 1% albumin (Sigma), 0.25% 2-mercaptoethanol (50**mM, Invitrogen), 0.12% glutamine (200 mM, Sigma), bFGF (20 ng/ml, Tebue), GDNF (10 ng/ml, Tebue), EGF (10 ng/ml, Tebue), gentamycin and metronidazole (100 µg/ml)]. After 6 days *in* *vitro* free-floating enteric neurospheres (EnNS’s) were abound, while differentiating neurons and glia cells at the bottom could easily be discriminated. The supernatant with EnNS’s and differentiation neuronal and glial cells was cultivated further. The EnNS’s cultures were maintained up to 40 days at 37°C in a humified atmosphere (5% CO_2_). Every three days, half of the volume was replaced with fresh medium.

### Rat and Human Enteric Neurospheres (EnNS’s) Differentiation

After five days *in vitro*, the floating EnNS’s were harvested and utilized either for differentiation experiments or for transplantation. Rat EnNS’s or dissociated single cells were suspended in extra cellular matrix (ECM) gel (Sigma) and plated on coverslips in 24 well plates (Becton Dickinson). The ECM gel plugs were allowed to gel at 37°C for several minutes and topped with cultured medium for differentiation [Neurobasal A (Gibco) supplemented with 2% B27+ (with vitamin A, Gibco), 1% albumin (Sigma), 0.25% 2-mercaptoethanol (50 mM, Invitrogen), 0.12% glutamine (200 mM, Sigma), bFGF (20 ng/ml; Tebue), GDNF (10 ng/ml, Tebue), Neurturin (10 ng/ml Tebue), gentamycin and metronidazole (100 µg/ml)]. After 48h *in vitro* the EnNS’s and single cell cultures were fixed with 4% formaldehyde for 10 min and stored in phosphate buffered saline (PBS).

### Generation of Rat Brain Slice Cultures

Eight days old SD rats were used for slice culture preparation. After decapitation the brains were dissected under sterile conditions [33]. The frontal pole and the cerebellum were removed and the brains were placed in preparation medium, containing minimum essential medium (MEM, Gibco) with 1% glutamax (Gibco), 0.45% glucose (Sigma), 2.5% HEPES 1M, 0.1 mg/mL streptomycin (Sigma), 100 U/ml penicillin (Sigma) at pH 7.35 and 4°C. Approximately one mm of the brain from rostral to caudal were removed and 400 µm thick slices were prepared with a vibratome (Leica). Four to six brain slices were obtained and used for further experiments. The slices were immediately transferred into cell culture inserts (Becton Dickinson, pore size 0.4 mm) into six well plates (Becton Dickinson) containing 1 ml culture medium per well [34]. Culture medium consists of 42 ml MEM (Gibco), 25 ml basal medium eagle (BME, Gibco), 25 ml normal horse serum (NHS; Gibco), 1 ml glutamax (Invitrogen), 1.5 ml glucose (45%, Sigma), 2.5 ml HEPES 1M (Sigma), 2 ml bicarbonate (7.5%, Invitrogen), 0.1 mg/ml streptomycin (Sigma), 100 U/ml penicillin (Sigma) at pH 7.30. The brain slices were incubated at 37°C in humified atmosphere with 5% CO_2_. Brain slices were hosted up to 5 days before transplantation.

### EnNS’s Transplantation

The EnNS’s were stained with a vital cell tracer (CFSE, Invitrogen) following the manufacturer’s protocol. Extracellular matrix proteins provide an important framework for the enteric microenvironment during the process of enteric neuronal differentiation [35]. To preserve normal cellular interactions in culture the stained EnNS’s were suspended in ECM (Sigma) for differentiation and cell transplantation experiments. ECM gel (2 µl) containing approximately 250 EnNS’s was transplanted into cerebral cortex lesions (200 µm diameter), which were punched into the slices. The brain slices with ECM gel-EnNS’s transplants were kept *in vitro* up to 4 days at 37°C, 5% CO_2_. Slices were fixed with 4% formaldehyde solution in phosphate buffered saline (PBS) for 24 hours.

### Immunohistochemistry and Immunocytochemistry

#### Human and rat EnNS’s

Differentiated EnNS’s or single cell cultures from human appendices (huEnNS’s) and rat intestine (rEnNS’s) were stained for Nestin, Sox2, ß-Tubulin III, Oct4, Nanog and TH.

The cultures were washed with PBS, followed by 45 min 0.5% triton X-100 and blocked in 10% normal goat serum for one hour. Primary antibodies for Nestin (Millipore, mouse-anti-Nestin [1∶200]), Nanog (R&D System, goat-anti-Nanog [1∶20], Sox2 (R&D System, maus-anti-Sox2 [1∶50]), Oct4 (Abcam, rabbit-anti-Oct4 [1∶200], ß-Tubulin III (Millipore, mouse-anti-Tubulin [1∶1000]) and TH (Chemicon, sheep-anti-TH [1∶500] were added and incubated over night at 4°C. The specimen were washed three times with PBS and incubated with secondary antibodies (Alexa® 488 anti-mouse-IgG, Alexa® 488 anti-rabbit-IgG, Alexa® 488 anti-goat-IgG or Cy3 anti-sheep-IgG Chemicon) for four hours at room temperature (RT). 4′, 6-diamidino-2-phenylindole (DAPI) (Sigma, 0.3 µg/ml) was used as nuclear stain.

After staining of rEnNS’s and single cells from rat dissociated rEnNS’s the protein expression for Nestin, Nanog, Sox2, Oct4, ß-Tubulin III and TH were quantified against the DAPI stained rEnNS’s and single cells from dissociated rEnNS’s (n = 3).

#### Human tissue

Human appendices were fixed in 4% formaldehyde in PBS and embedded in paraffin. Adjacent sections of 10 µm were cut on a microtome (Leica). After dewaxing and rehydrating, the sections were washed in 0.05% tween and after antigen retrieval in citrate buffer (30 min, 95°C, pH 6.0) tissue were allowed to cool for 30 min at RT. After blocking with normal goat serum the tissues were incubated in an antiserum raised against the neuronal marker PGP 9.5 (Dako, [1∶500]) or sheep-anti-TH (Chemicon, [1∶500]) over night at 4°C. The detection of PGP 9.5 antigen was carried out with the EnVision+ System-HRP for use with rabbit secondary antibodies (Dako). DAB was used as a chromogen to localize the peroxidase in our tissue sections whereas the detection of TH was carried out with Vectastain Elite ABC kit (Linaris) according to the manufacture’s protocol. After the DAB reaction, the sections were dehydrated and mounted in Neo-Mount (Merck).

#### Brain slices with transplanted EnNS’s

The rat brain slices were stained for TH and ß-Tubulin III, GFAP and Nestin. The slices were washed with PBS, followed by 45 min 0.5% triton X-100 and blocked in 10% normal goat serum for one hour. Primary antibodies for Nestin (Millipore, mouse-anti-Nestin [1∶200]), ß-Tubulin III (Millipore, mouse-anti-Tubulin [1∶1000]), GFAP (Dako, rabbit-anti-GFAP [1∶500]) and TH (Chemicon, sheep-anti-TH [1∶500] were added and incubated over night at 4°C. The slices were washed three times with PBS and incubated with secondary antibodies (Alexa® 546 anti-mouse-IgG, Alexa® 546 anti-rabbit-IgG or Cy3 anti-sheep-IgG, Chemicon) for four hours at RT. DAPI (Sigma, 0.3 µg/ml) was used as nuclear stain.

#### Real-time PCR analysis of gene expression

Total cellular RNA from EnNS’s and neuronal cells was extracted using an Isolate RNA Mini Kit (Bioline, Luckenwalde, Germany) following the manufacturer’s protocol. Contaminating genomic DNA was digested with DNAse I (Invitrogen, Karlsruhe, Germany). RNA concentration was determined spectrophotometrically with the infinite M200 micro plate reader (Tecan, Mainz-Kastel, Germany) and RNA quality assessment was tested with RNA 6000 Nano Kit (Agilent Technologies, Waldbronn, Germany) and the Agilent Bioanalyzer 2100 (Agilent Technologies, Waldbronn, Germany).

BioScript TM was used to generate cDNAs (Bioline, Luckenwalde, Germany). For real time PCR the SensiMixSYBR Low-Rox Kit (Bioline, Luckenwalde, Germany) was used on a MX3005 (Stratagene, Amsterdam, Netherlands). Glyceraldehyde-3-phosphate dehydrogenase (GAPDH) was used as an internal standard. The PCR conditions were as follows: initial denaturation 10 min, 95°C, 40 cycles of denaturation, 30 sec., 95°C; annealing, 30 sec., 56°C (nestin, GAPDH); 30 sec., 72°C. The Primer sequences (F: forward; R: reverse) were as follows: human Nestin (F: 5′-CTCCAAGAATGGAGGCTGTAGGAA-3′, R: 5′-CCTATGAGATGGAGCAGGCAAGA-3′) and human GAPDH (F: 5′-GCACCGTCAAGGCTGAGAAC-3′, R: 5′-TGGTGAAGACGCCAGTGGA-3′), from [36]. For rat Nanog (F: 5′-TTGGAACGCTGCTCCGCTCC-3′, R: CGCCTGGCTTTCCCTAGTGGC-3′), rat Sox2 (F: 5′-ACTAATCACAACAATCGCGGCGGC-3, R: 5′-GACGGGCGAAGTGCAATTGGGA-3′) rat Oct4 (F: 5′-GGAGGGATGGCATACTGTGGACCT-3′, R: 5′-TCCTGGGACTCCTCGGGACTAGG-3′) from [37] and rat GAPDH (F: 5′-GTATGACTCTACCCACGGCAAGT-3′, R 5′-TTCCCGTTGATGACCAGCTT-3′) from [38] were used. Data were normalized for GAPDH mRNA expression using the delta-delta-CT method. Data is expressed as the mean ± SEM and tested for statistical significance using student’s t-test. The results were considered significant if the probability of error (P) was *P<0.05.

#### Microscopy

EnNS’s and differentiated cells on coverslips, tissue and brain slice were visualized using a BIOREVO BZ-9000 microscope (Keyence) and the analysis software BZ-II Analyzer (Keyence). Image processing was performed with the open source program Gimp.

## Results

### Potent ENS Precursor Cells are Derived from Enteric Plexus

**Figure 1:**
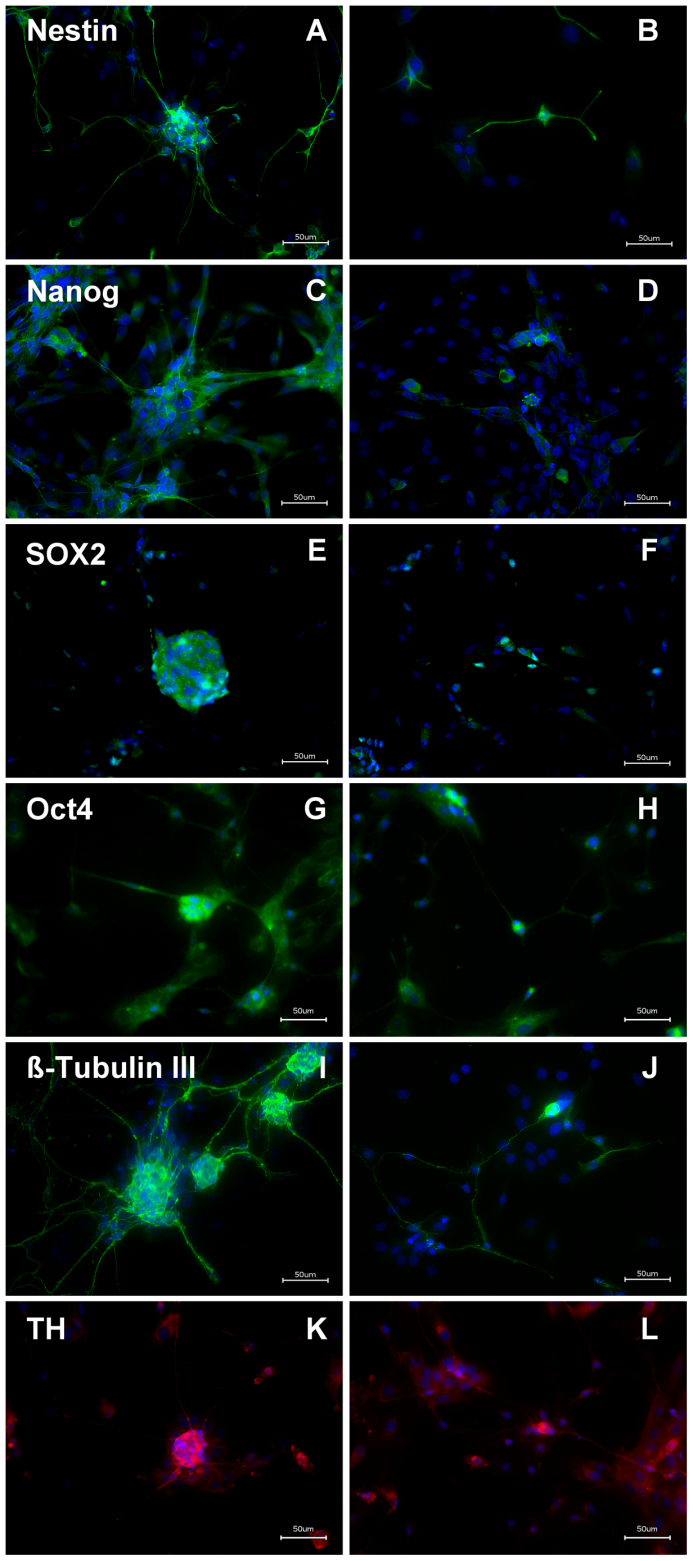
Immunohistochemistry from rat EnNS’s of the myenteric plexus cultivated in extracelluar matrix. Rat EnNS’s (**A, C, E, G, I**) and single cells from dissociated rEnNS’s (**B, D, F, H, J, L**) were fixed and stained after 48 hours *in vitro* with various stem cell (**A–H**) and neuronal markers (**I–L**). The cells were immunostained with the markers for Nestin (**A, B**), Nanog (**C, D**), Sox2 (**E, F**) Oct4 (**G, H**), ß-Tubulin III (**I, J**) and TH (**K, L**). DAPI was used as a nuclear stain.

The free floating EnNS’s from rat culture preparations were dissociated and cultivated. Within 48 hours the dissociated neural spheres and isolated neural cells developed an intricate network with glia, neurons and interconnecting fibers, as seen in primary enteric cultures before. Immunostaining with Nestin, Nanog, Sox2, Oct4, ß-Tubulin III, and TH were positive in different cell types (Fig. 1 A–L). The expression of Nanog, Sox2 and Oct4 in rEnNS’s was confirmed in the real-time PCR experiments (Table S1).

**Figure 2:**
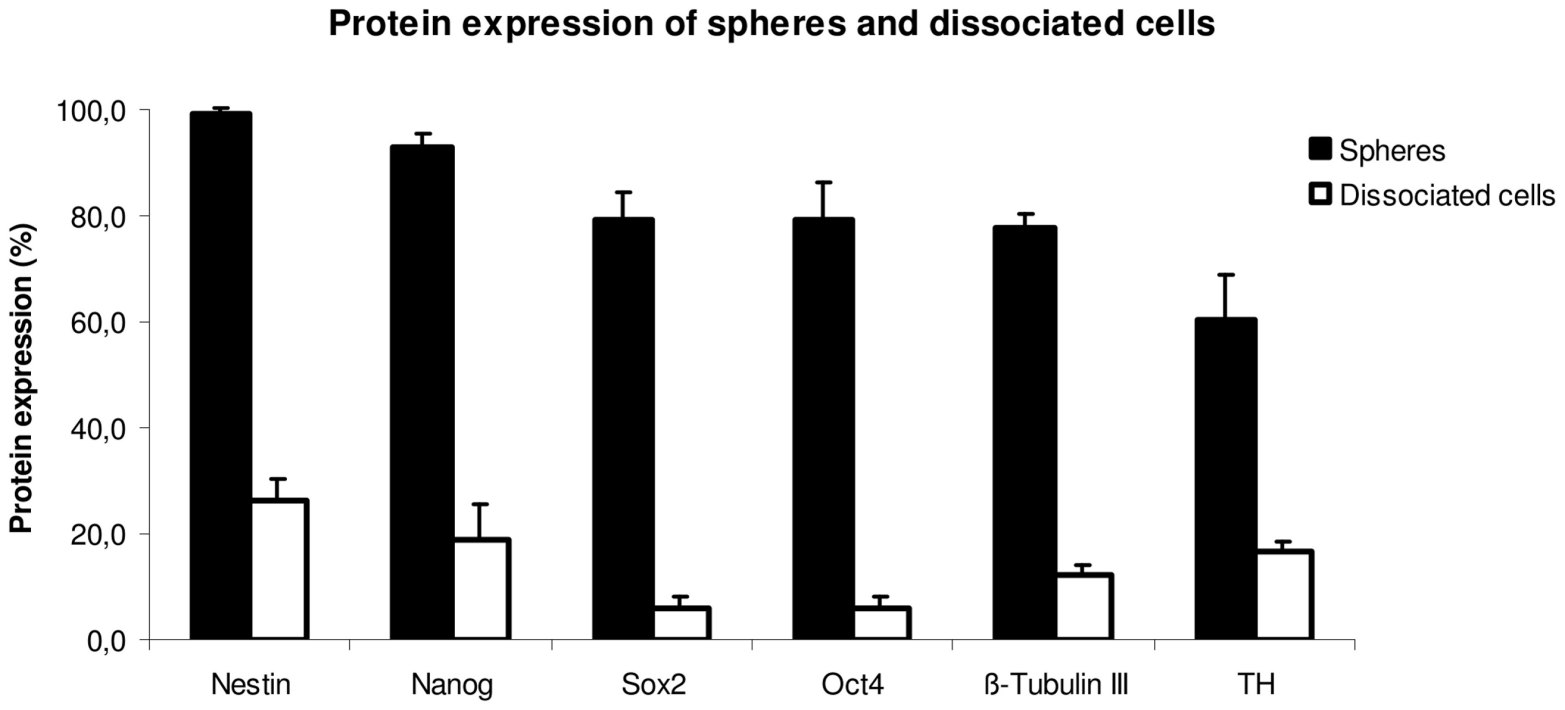
Protein expression of EnNS’s and dissociated cells. The protein expression of rEnNS’s and single cells from dissociated rEnNS’s after 48 hours *in vitro* were quantified for Nestin, Nanog, Sox2, Oct4, ß-Tubulin III and TH.

The neural spheres and isolated cells revealed stem cell characteristics as well as qualities of neuronal and glial cells. The neural spheres had a positive immunostaining for the neuroepithelial stem cell marker Nestin (Fig. 1 A, B) and ß-Tubulin III (Fig. 1 I, J). Nestin positive cells can be regarded as neural progenitor cells [39], [40], while ß-Tubulin III positive cells are possibly differentiating neuronal cells [41]. Furthermore the generated EnNS’s were also positive for Nanog (Fig. 1C, D), Sox2 (Fig. 1 E, F) and Oct4 (Fig. 1 G, H). Sox2 as well as Oct4 is a transcription factor essential for maintaining self-renewal or pluripotency of undifferentiated embryonic stem cells [42]. Together with Oct4 it forms a heterodimer and binds DNA [43], [44]. Nanog is a protein expressed in embryonic stem cells and is together with Sox2 and Oct4 thought to be a key factor in maintaining pluripotency [45], [46]. The presence of TH positive cells (Fig. 1 K, L) demonstrates that these EnNS’s derived from the gastrointestinal tract might give rise to a dopaminergic neuronal cell fate. The statistical evaluation revealed an abundant protein expression, while in the dissociated cell preparation the protein expression decreased significantly (Fig. 2). The rEnNS’s demonstrated an abundant expression of Nestin, Nanog, Sox2 and Oct4 while in the dissociated single cells only 6% of the cells expressed pluripotence genes (Sox2, Oct4) and 26.3% (Nestin) respectively 19% (Nanog) demonstrated stem cell characteristics. In contrast the TH expression was to be seen in 16.7% of the dissociated neural cells.

### Rat Derived EnNS’s (rEnNS’s) Transplantation

**Figure 3:**
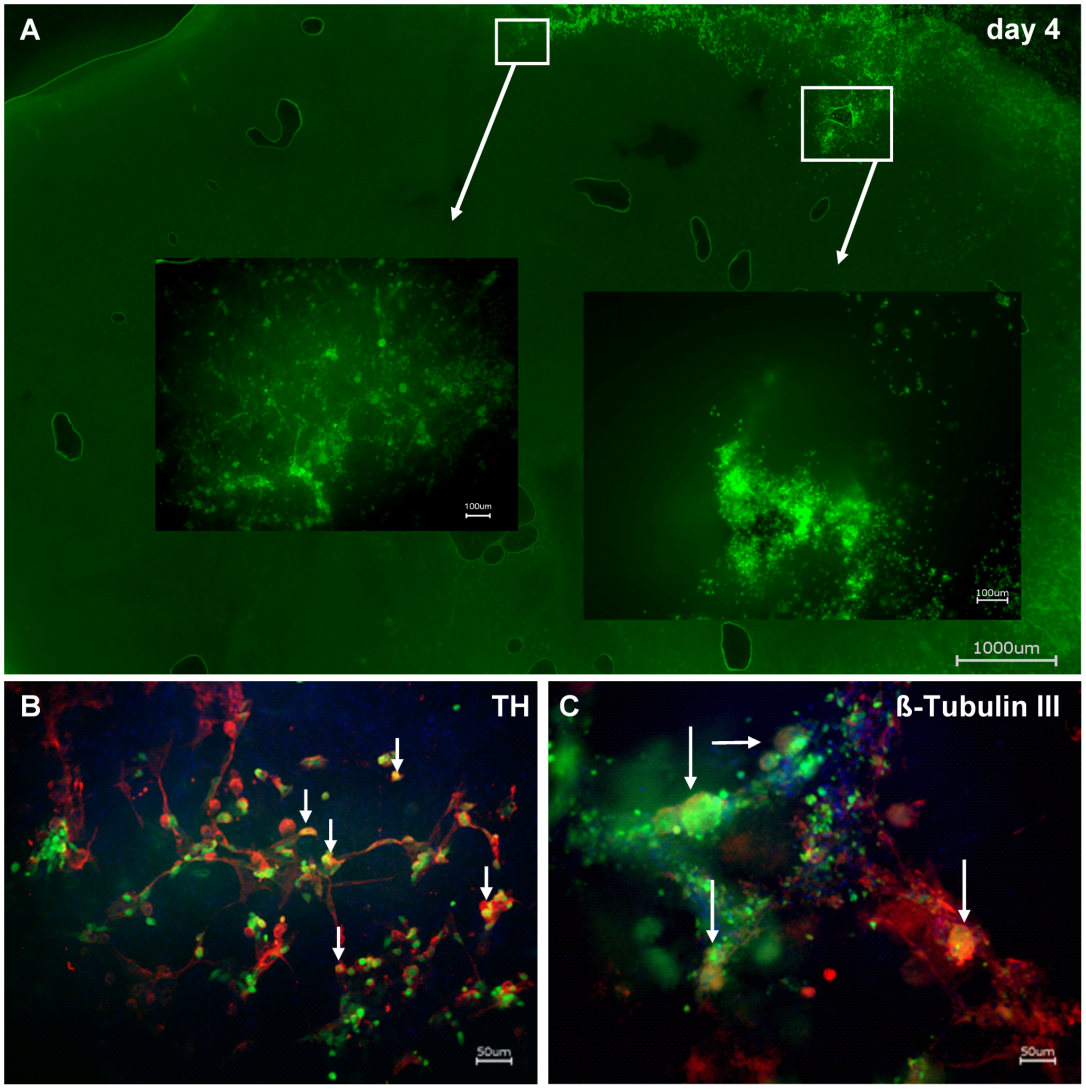
Transplanted rat EnNS’s in rat brain slices. rEnNS’s were stained with CFSE and transplanted into the cortical layer of a rat brain slice. (**A, B** and **C**). Within for days the surviving transplanted cells migrated into the cortical layers. At two distant regions (white squares) the transplanted cells are shown in a magnification. Rat brain slice with CFSE labelled rEnNS’s (green) transplants were immunostained with TH (red) (**B**) and ß-Tubulin III (red) (**C**). For single cells a co-labelling for CFSE-stained transplants and TH (**B**, arrowheads) as well as for CFSE-stained transplants and ß-Tubulin III was seen (**C**, arrowheads). DAPI was used as a nuclear stain.

EnNS’s from the rat intestine were transplanted into rat brain slices. After injury the transplanted CFSE marked rEnNS’s could be clearly seen within the brain slice. Microscopic tracking of the transplants in brain slice culture was possible for up to five days; beyond this the clear detectable CFSE signal vanished due to cell growth and migration into the brain slice (3 A–C). *In vitro* transplanted rEnNS’s survived for more than four days, differentiated and migrated into rat brain slices (Fig. 3A). Microscopic observation of the transplants revealed neurite outgrowth and network formation, while the transplanted cells also formed interconnections with the surrounding tissue.

Immunohistochemical staining with TH and ß-Tubulin III revealed a strong signal for all staining. For ß-Tubulin III and TH a co-labeling in transplanted neural cells could be demonstrated (Fig. 3C, D). This finding proves the in situ differentiation of the transplanted cells.

### Human Tissue

Appendices from 81 patients (59 children, aged 1 to 12 years; 29 girls, 30 boys and 22 adults, aged 16 to 67 years; 13 female, 9 male patients) were obtained and prepared for huEnNS’s culture and immunostaining. All children and 17 adult patients had appendectomy due to inflammation; the other patients underwent a colectomy because of adenocarcinoma. All together 55 appendices could be processed within 24 hours, while 26 specimens were stored in MEM over 24 hours and were used for immunostaining only.

### Immunostaining of Human Tissue

**Figure 4:**
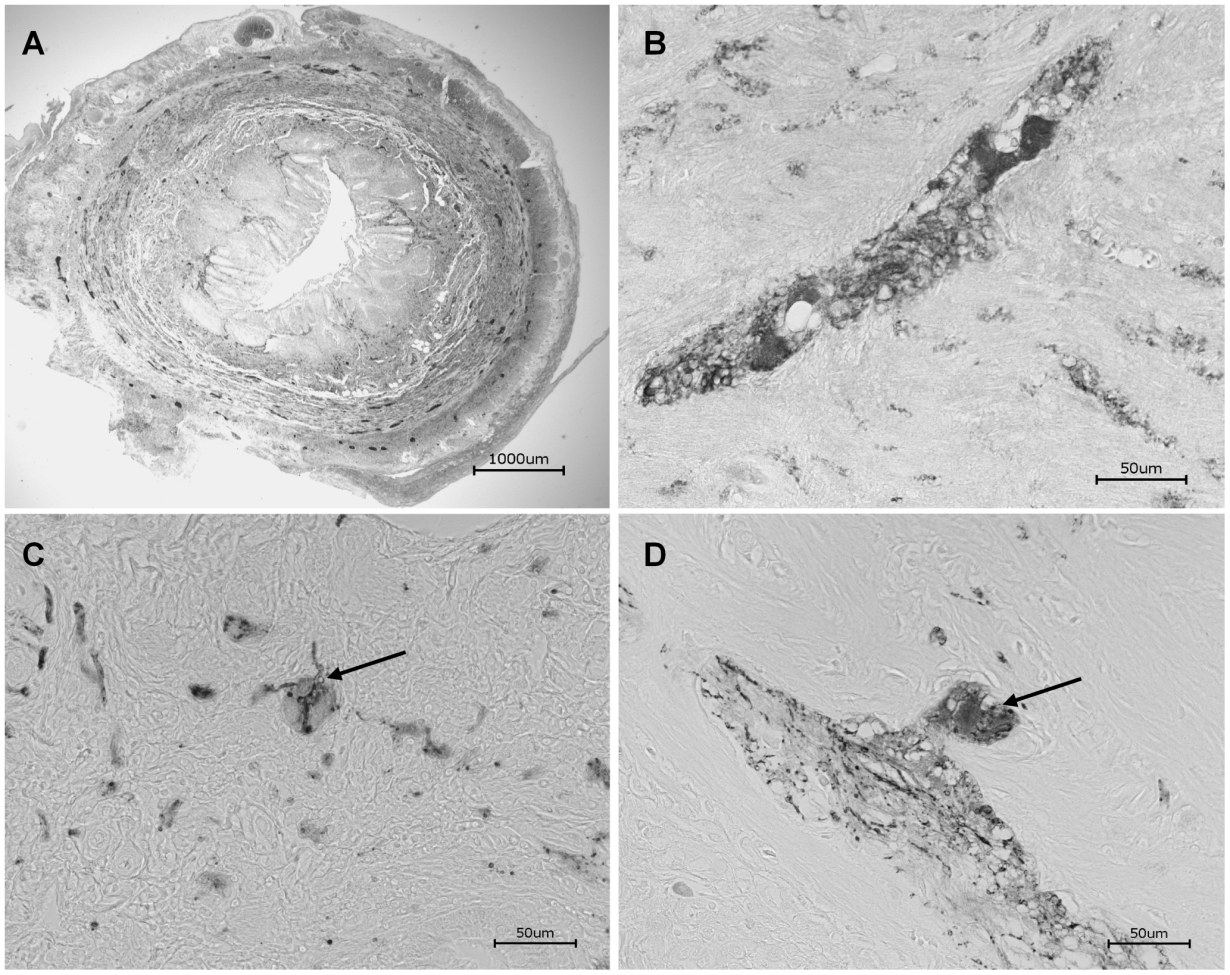
Immunohistchemistry of human appendix tissue. A whole appendix (**A**) is stained with the pan neuronal marker PGP9.5 and in the enteric plexus (**B**). TH positive cells could be demonstrated in the submucous (**C**, arrowheads) and myenteric (**D**, arrowheads) plexus.

The general enteric stem cell niches were first studied in human tissues (Figure 4 A–D). To identify neural cells protein gene product (PGP 9.5) [47] and TH were immunolabeld [32].

TH as the rate-limiting enzyme for catecholamine biosynthesis is present in all central and peripheral neurons, which utilize the neurotransmitters dopamine, epinephrine and norepinephrine [48]. Therefore the appendices were also stained for TH [49]. In all stained tissues the markers demonstrated a clear signal within the submucous and myenteric plexus (Fig. 4 A–D). Especially TH positive neurons could be found (Fig. 4C, D), suggesting that these neurons are progeny from neural crest derived cells that differentiate into catecholaminergic neurons.

### Human Enteric Neurospheres (huEnNS’s) are Derived from Human Appendix

**Figure 5:**
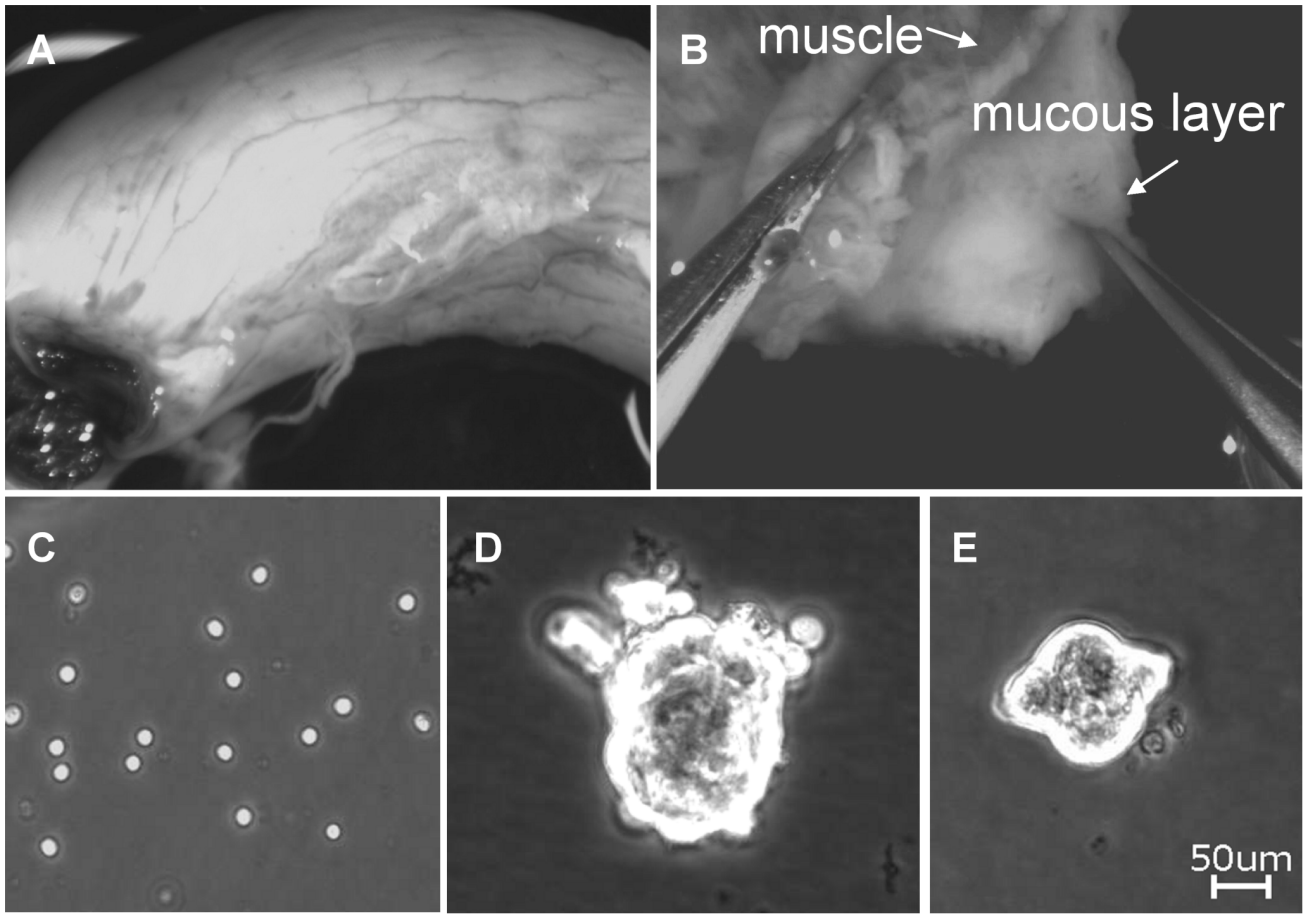
EnNS’s isolated from the human appendix (huEnNS’s). The human appendix as used (**A**). The appendix was separated in the mucosa and muscular layer (arrowheads) under visual control (**B**). After the preparation procedure single cells could be isolated (**C**). Depending on the donors age within 5 to 15 days EnNS’s could be detect. Sphere from the submucous (**D**) and the myenteric plexus (**E**).

According to the previously described method [50], [51] it was possible to isolate submucous (SMP) and myenteric plexus (MP) from most of the human appendices (Fig. 5A, B). After incubating the samples in a collagenase solution usually only single cells could be isolated (Fig. 5C). In culture first adherent neurons and glia could be detected. While the differentiated neuronal and glial cells disappeared *in vitro*, another round shaped cell type started to proliferate within 5–7 days, forming growing cell clusters. These huEnNS’s were free floating cell clusters without processes (Fig. 5D, E). In the culture preparation with appendices from children younger than 5 years first huEnNS’s could be seen after three days while cultures from patients older than 65 years first huEnNS’s appeared after ten days. Myenteric plexus preparation yielded 378±50 huEnNS’s per preparation, while the submucous plexus yield was 518±50 huEnNS’s. In general the submucous plexus preparation showed an increased growth potential but was more often prone to bacterial overgrowth, especially after storage or transportation times of more than 12 hours.

Secondary and tertiary human enteric nervous system neurospheres can be exponentially expanded by repeated dissociation without deficiency in their differentiating potential [52]. The same characteristics persisted in our cultures up to 40 days or until we used them in other experiments. Taken as a whole 62% (34 appendices) of the culture preparations lead to successful huEnNS’s generation, while bacterial overgrowth impeded it in 27% (15 appendices) and for 11% (6 appendices) of the harvested plexus preparation no reason for the failure was obvious.

### Potent ENS Precursor Cells are Derived from Human Enteric Plexus

**Figure 6:**
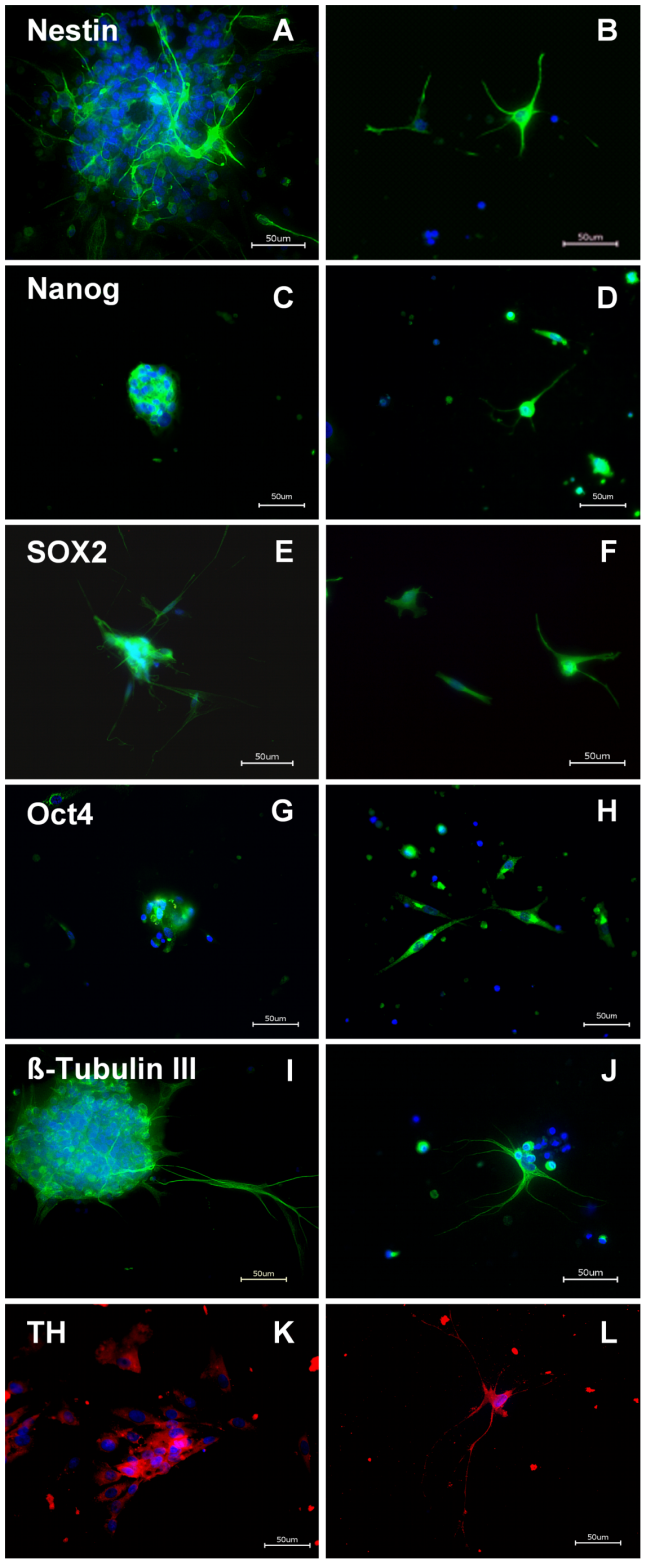
Immunochemistry of human appendix EnNS’s of the submucous plexus, 48h *in vitro* in extracelluar matrix. huEnNS’s (**A, C, E, G, I**) and single cells from dissociated huEnNS’s (**B, D, F, H, J** and **L**). huEnNS’s and single cells were stained with the same stem cell and neuronal markers as used in the rat. Nestin (**A, B**), Nanog (**C, D**), Sox2 (**E, F**) and Oct4 (**G, H**) as stem cell markers, ß-Tubulin III (**I, J**) and TH (**K, L**) for neuronal cells. DAPI was used as a nuclear stain.

**Figure 7:**
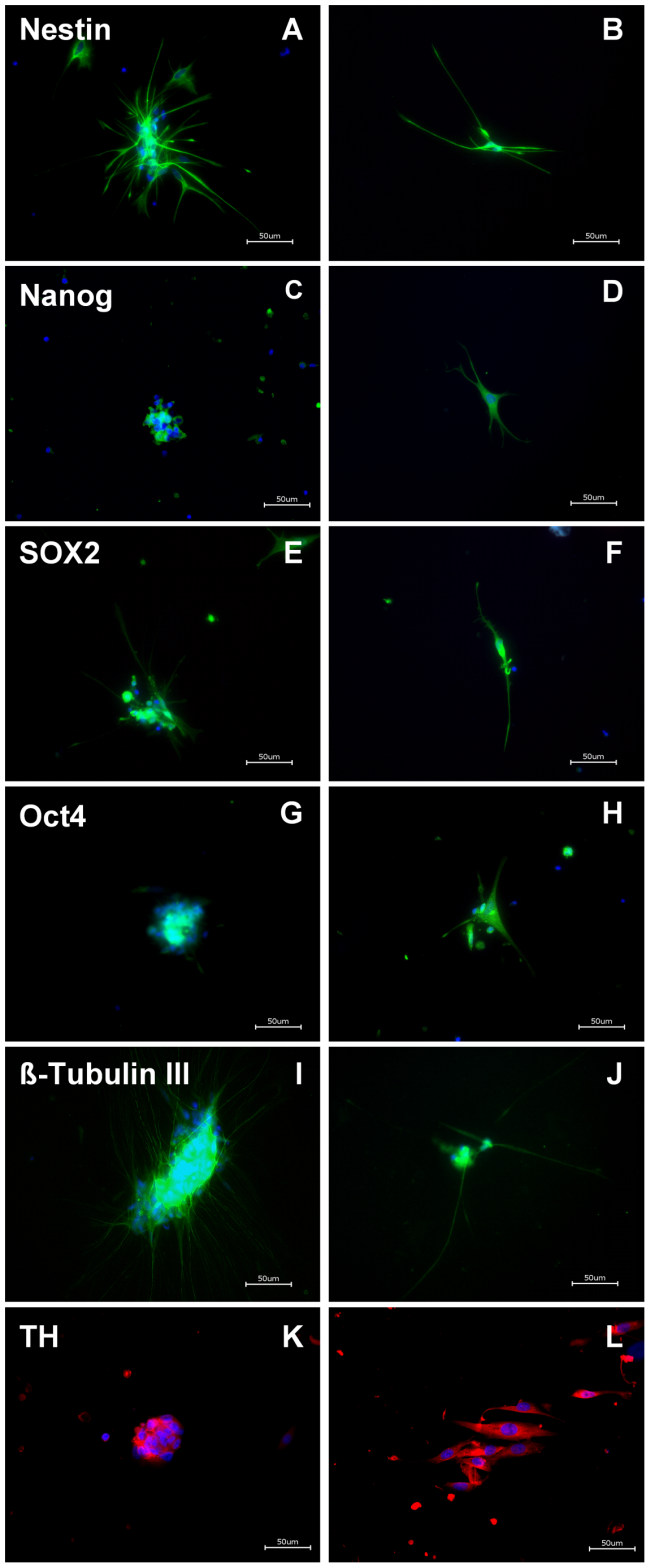
Immunohistochemistry of human appendix EnNS’s of the myenteric plexus, 48h *in vitro* in extracelluar matrix. huEnNS’s (**A, C, E, G, I**) and single cells from dissociated huEnNS’s (**B, D, F, H, J** and **L**). huEnNS’s and single cells were stained with the same stem cell and neuronal markers as used in the rat. Nestin (**A, B**), Nanog (**C, D**), Sox2 (**E**, **F**) and Oct4 (**G, H**) served as stem cell markers, ß-Tubulin III (**I, J**) and TH (**K, L**) for neuronal cells. DAPI was used as a nuclear stain.

The free floating huEnNS’s from both, MP and SMP culture preparations were harvested, dissociated and cultivated for differentiation (Fig. 6 and 7). Planted huEnNS’s showed neurite outgrowth within 12 hours, which led to network formation. In cultures of dissociated huEnNS’s cells, neuronal cells with interconnecting nerve fibers and glia cell morphology could be discriminated within 48 hours.

Immunostaining with Nestin, Nanog, Sox2, Oct4, ß-Tubulin III and TH were positive in different cell types (Fig. 6 A–L: SMP, Figure 7 A–L: MP).

The neural spheres and isolated cells from MP and SMP also revealed stem cell characteristics as demonstrated by the immunoreactivity for Nestin (Fig. 6A, B: SMP; Fig. 7 A, B: MP), Nanog (Fig. 6C, D: SMP, Fig. 7C, D: MP), Sox2 (Fig. 6E, F: SMP, Fig. 7E, F: MP) and Oct4 (Fig. 6G, H: SMP, Fig. 7 G, H: MP). Furthermore the generated huEnNS’s were also positive for ß-Tubulin III (Fig. 6I, J: SMP, Fig. 7 I, J: MP) and TH (Fig. 6 K, L: SMP, Fig. 7 K, L: MP) confirming their neuronal characteristic and the potential to give rise to a dopaminergic neuronal cell fate. Induced pluripotent stem cells (iPSC’s) were used as positive control for Nanog and Oct4 (Figure S1). Real-time PCR analysis also revealed a significant increase of Nestin in SMP (7.86±4.23) and MP (4.62±1.67) spheres compared to differentiated neurons that served as control (Table S1).

### Human EnNS’s Transplantation

**Figure 8:**
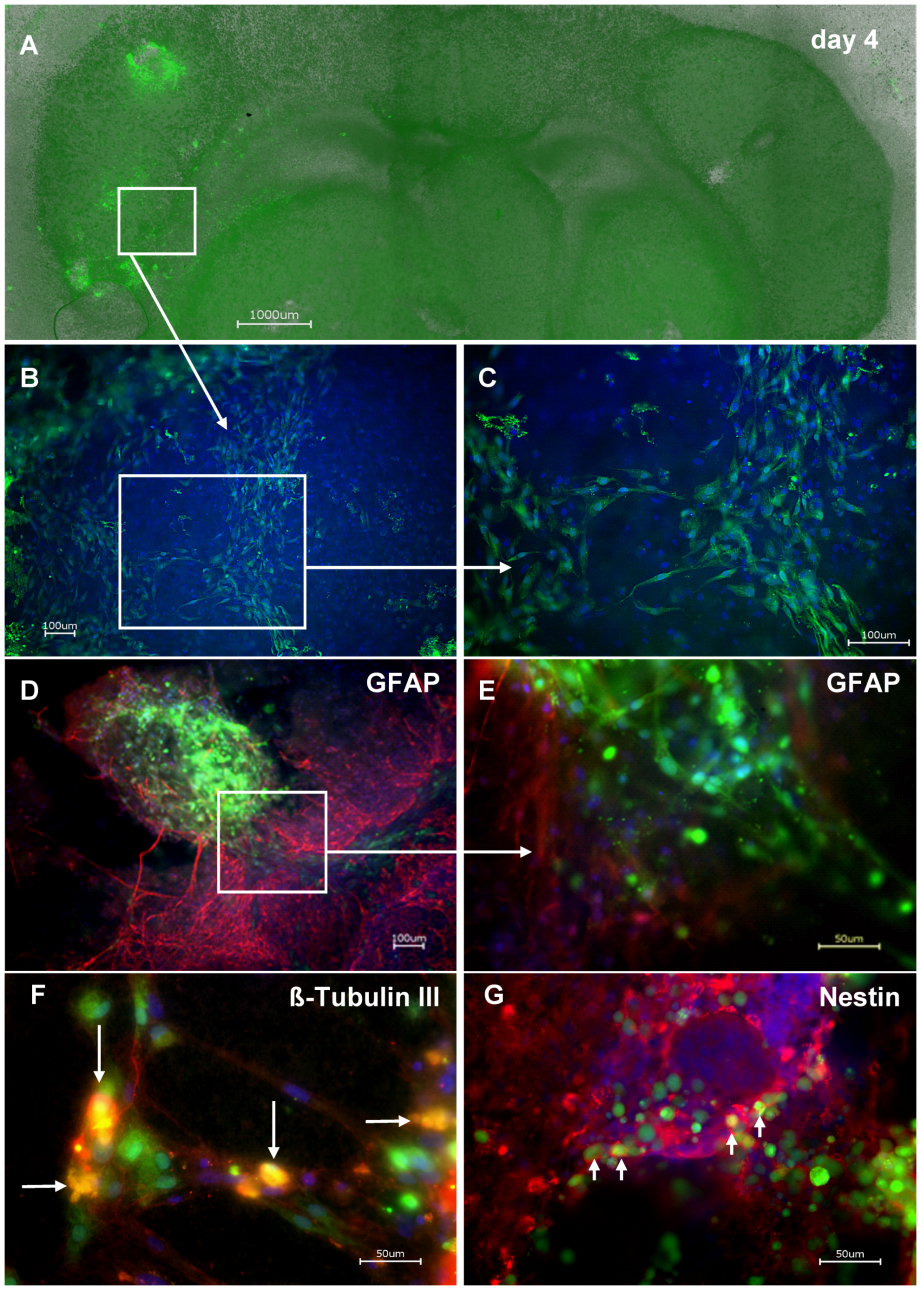
Transplanted human EnNS’s in rat brain slices. huEnNS’s from the human appendix were stained with CFSE (green) and then transplanted in the cortical layer of a rat brain slice. (**A**) Complete rat brain slice after 4 days of transplanted human EnNS’s. The area of the transplant is seen in the cortical layer, while EnNS’s, which migrated over four days into the brain slice are seen in the inner layers (**B, C**) Parts of growing transplanted and differentiated spheres in the rat brain slice. Cells that migrate from the transplant (white square, **A**) are shown in magnification (**B, C**). With the CFSE fluorescence single cells can be discriminated and the intricate network formation is explicit mapped (**C**). Rat brain slice transplanted with CFSE labelled neuronal spheres (green) from the human appendix were immunostained with (**D, E**) GFAP and DAPI, (**F**) ß-Tubulin III and DAPI and (**G**) Nestin and DAPI. A co-labelling for CFSE labelled and ß-tubulin III positive cells (F, yellow cells, arrowheads) as well as for CFSE labelled and nestin positive cells (**G**, yellow-green cells, arrowheads) could be shown.

As a proof of principal huEnNS’s were transplanted into rat brain slices. After injury the transplanted CFSE marked huEnNS’s could be clearly seen within the brain slice. During cultivation over at least four days transplanted huEnNS’s derived from SMP and MP survived, differentiated and migrated into rat brain slices (Fig. 8). Microscopic observation in culture revealed neurite outgrowth and network formation. The transplanted cells also formed interconnections with the surrounding tissue.

After four days *in vitro* the brain slices containing human derived EnNS transplants were fixated and stained against glial fibrillary acidic protein (GFAP), ß-Tubulin III and Nestin (Fig. 8 A–G). In the surrounding neuronal tissue indigene GFAP positive glial cells recreated interconnections, as described for reactive gliosis [7]. But at the transplant/host border there were also transplanted EnNS’s to be seen that passed across the astroglial border. For ß-Tubulin III (Fig. 8F) and Nestin (Fig. 8G) a co-labeling for transplanted and indigene neuronal cells could be demonstrated, similar to the rat cultures.

## Discussion

In this presented work we demonstrated the ability to cultivate and differentiate enteric neurospheres (EnNS’s) from human appendix tissue of all ages. The appendix as an easy accessible part of the intestine can be used for neural stem cell generation. With minimal invasive surgical techniques the appendectomy is not harmful, even in diseased patients [53]. In ischemic stroke multiple cell types like neurons, glial cells and blood vessels are lost while in neurodegeneration predominantly neuronal subpopulations are affected. Ideally, the cell replacement therapy involves comprehensive replacement or induction of various cell types. The enteric nervous system with its intimate contact to luminal contents of the gut is in contrast to the CNS permanent challenged. Due to dietary habits, mechanical bowel movements and inflammatory responses, the ENS has to handle tissue damage or neuronal cell loss continuously [54], [55]. Replacement of lost neuronal and glia cells might be one of the concepts for the lifelong intestinal plasticity [28], [56], [57]. The ENS does not only host an active stem cell niche with neuronal and glial precursors it is also able to support vascular regeneration [58]. In the human CNS blood vessels originate from extrinsic cell populations, which interact with glia and neurons to establish the blood brain barrier and control cerebrovascular exchanges. Furthermore neurovascular interactions play an important role in adult neurogenic niches as indicated by intimately associated neural stem cells with blood vessels [59]. In an actual study we could demonstrate that co-cultivation of EnNS’s with mesenteric vascular cells (MVC’s) facilitates neuronal regeneration and tube formation [58]. The generated neurospheres and/or dissociated cells showed characteristics of neural stem cells (Nestin, Nanog, Sox2 and Oct4 positive), differentiating and mature neurons (ß-Tubulin III positive, Tyrosine Hydroxylase (TH) positive). These tremendous Nestin expression has been described in enteric stem cells from rat embryonic gut [51], substantiating the presumption that the derived EnNS’s are pluripotent and can be differentiated into neuronal and glial cells. In contrast, only 6% of the dissociated cells expressed pluripotency genes, which gives evidence for a smaller cell population in the enteric nervous system that remains pluripotent. Such cells have been described in the adult mouse [60]. Hence the enteric nervous system harbors diverse kinds of neuronal stem cells that give rise to subtypes of neuronal and glial cells. In particular the verification of TH positive neurons in the appendix, together with the expression of several key molecules for pluripotency, make these cells enormously valuable for regenerative cell therapy in neurological disorders such as Parkinson’s disease [61]. Due to the fact, that several of the pluripotency genes are already expressed, these cells might easily be transformed into induced pluripotent stem cells (iPSC’s) with a little amount of reprogramming. Furthermore as the enteric neurons are neural crest derived [62] it is very likely that TH positive neurons in central and enteric nervous system are progeny from the same source, suggesting that enteric neurons might smoothly integrate and reconstitute neuronal function in the CNS. Enteric precursors with a chemical coding and transmitter spectrum similar to the ENS are able to support host neurogenesis. Moreover, the various enteric neuronal populations also comprise cholinergic, serotonergic and catecholaminergic phenotypes [61].

In our *in vitro* experiments host astrocytes promoted regeneration as seen by the accumulation of GFAP positive cells along the transplant/host border without entering the transplant. GFAP is expressed in reactive astrocytes that rebuilt the blood brain barrier [7], [63], [64]. As the GFAP reactive host cells did not enter the transplanted enteric neurons, the formation of a glial scar has to be assumed in our transplantation experiments. On the other hand a great proportion of transplanted huEnNS’s migrate across the astroglial lining, which indicates a promoted regeneration and neuronal regeneration is also described to occur beyond the glial scar [65].

Beside the astrocyte response our transplants demonstrated further enormous potential for neurogenesis. Plasticity in the adult CNS depends the constitutive production of neuronal cells and a small proportion of neuronal stem cells have been located in neurogenic areas such as the subventricular zone [66]–[68]. While in neurodegenerative disorders like Parkinson’s disease alterations in the subventricular zone are seen [17], [69]. ENS precursor cells might be used to create a supportive environment in these weakened neurogenic areas. In Parkinson’s disease (PD) the progressive loss of dopaminergic neurons in the CNS and the presence of cytoplasmic eosinophilic inclusions, the Lewy bodies, are pathological hallmarks [70], [71]. The ENS of patients with PD also presents Lewy bodies within its enteric plexus [72] with decreased dopamine neurons [73] but TH immunoreactive neurons were not altered [74], [75]. A new studies even states that the ENS is not at all, or much less involved in alterations as presumed [76].

Studying the ENS for cell replacement therapies might add deeper insights in barriers that have to overcome. In cerebral ischemic stroke an ischemic cascade follows the reduced blood supply thus affecting potentially salvageable tissue [77]. The microenvironment in the gut has continuously to cope with changing cellular interactions, impaired blood supply and inflammation [78]. For instance, the gastrointestinal tract also has a blood ENS barrier and enteric glial cells are key regulators of intestinal barrier functions [79], [80]. In a rat model transplantation of enteric glia into the spinal chord accelerated blood brain barrier formation [81].

While mature ENS cells from rodents have been used for central nervous tissue repair [82], this is the first study which demonstrates the potential of ENS derived neural stem cells from both rodent and human gut and might lay the foundation of future neural stem cell strategies based on the use of ENS derived neural stem cells. Self renewal and differentiation, the attributes of an ongoing neurogenesis proceed in the adult ENS [28], making it an excellent autologous neural stem cell source. Furthermore the enteric neurospheres are already primed to a neuronal cell fate and should be redifferentiated to iPSC’s with less extensive manipulation. *In vivo* studies with iPSC’s derived from enteric neuronal tissue will be performed in the near future. All together the ENS seems to be the perfect source not only for catecholaminergic or cholinergic neurons, but also for different glial cell types as just demonstrated [83] to be used for blood-brain barrier repair or Multiple Sklerosis.

## Supporting Information

Figure S1 **Oct4 and Nanog staining.** Induced pluripotent stem cells (iPSC’s) were used as positive control. IPSC’s were stained with either with Oct4 (Abcam, rabbit-anti-Oct4) or Nanog (R&D System, goat-anti-Nanog). DAPI was used as a nuclear stain. In all preparations the signal is explicitly mapped.
(TIFF)

Table S1 **RT-PCR in rEnNS’s.** Expression of the stem cell markers Nanog, Sox2 and Oct4 in rat EnNS’s (n = 6).
(DOCX)
