# Supplementary figures and images for: The Human Gastrointestinal Tract, a Potential Autologous Neural Stem Cell Source
Source: PLoS One. 2013 Sep 4;8(9):e72948. doi: 10.1371/journal.pone.0072948 (PMC3762931; doi:10.1371/journal.pone.0072948)

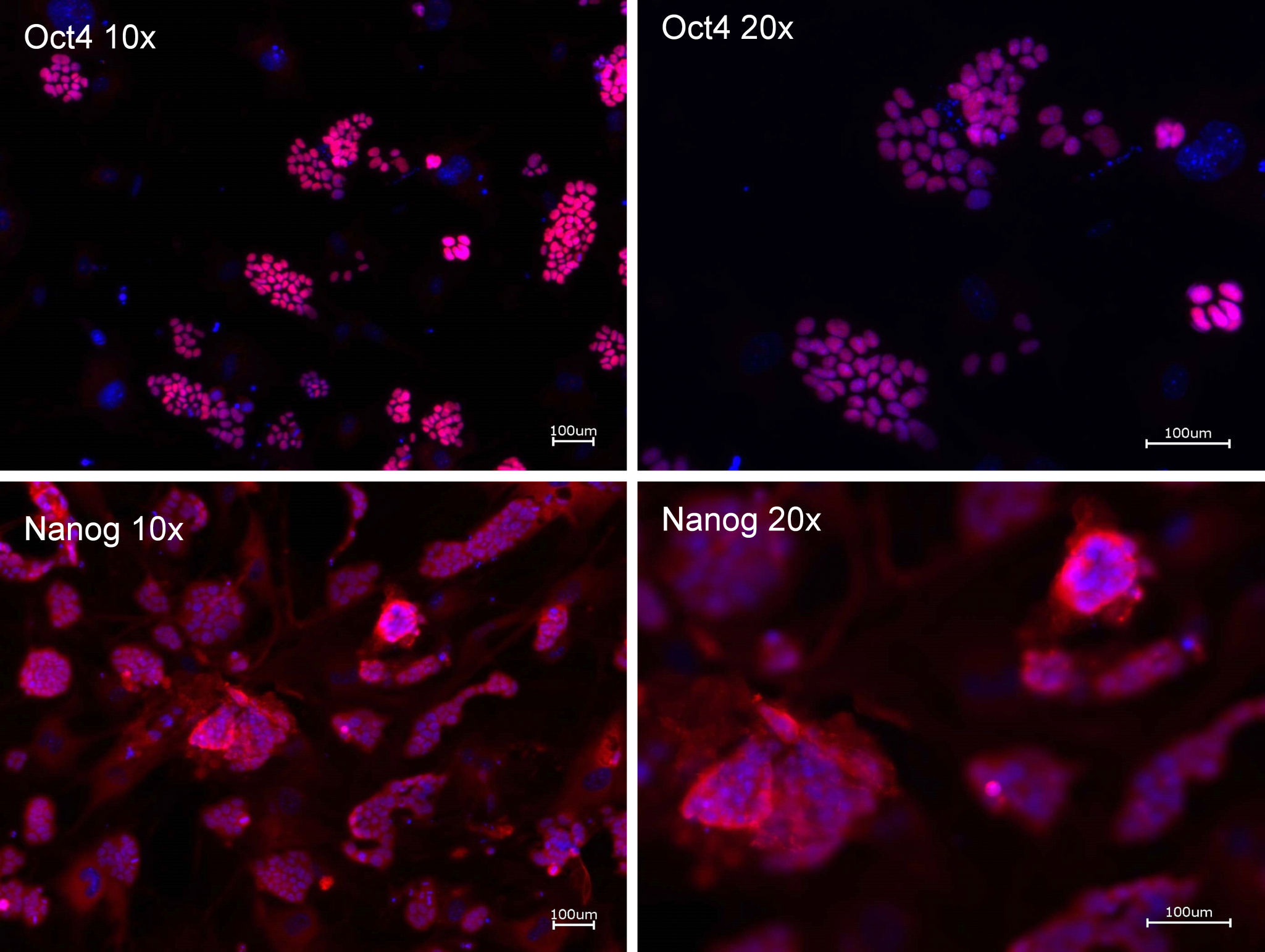

Supplement: Figure S1 — Oct4 and Nanog staining. Induced pluripotent stem cells (iPSC’s) were used as positive control. IPSC’s were stained with either with Oct4 (Abcam, rabbit-anti-Oct4) or Nanog (R&D System, goat-anti-Nanog). DAPI was used as a nuclear stain. In all preparations the signal is explicitly mapped. (TIFF) [file pone.0072948.s001.tiff]
