# Supplementary material for: The Human Gastrointestinal Tract, a Potential Autologous Neural Stem Cell Source
Source: PLoS One. 2013 Sep 4;8(9):e72948. doi: 10.1371/journal.pone.0072948 (PMC3762931; doi:10.1371/journal.pone.0072948)
Supplement: Table S1 — RT-PCR in rEnNS’s. Expression of the stem cell markers Nanog, Sox2 and Oct4 in rat EnNS’s (n = 6). (DOCX) [file pone.0072948.s002.docx]

**Table S1 RT-PCR in rEnNS’s**

Expression of the stem cell markers Nanog, Sox2 and Oct4 in rat EnNS’s (n=6)

|  | **Nanog** | | **Sox2** | | **Oct4** | |
| --- | --- | --- | --- | --- | --- | --- |
|  | Mean | SEM | Mean | SEM | Mean | SEM |
| rEnNS’s | 1.24 | 0.1885 | 6.12 | 1.809 | 31.00 | 12.24 |
